# The subacromial bursa in rotator cuff injury: from pathological contributor to regenerative resource?

**DOI:** 10.3389/fbioe.2026.1878354

**Published:** 2026-07-13

**Authors:** Xiong Wang, Qing Gu, Feifei Zhang, Lu Li, Jinqiang Zhang, Wenqiang Wei, Shuming Zi, Song Chen

**Affiliations:** 1 Department of Sports Medicine, Tongji Hospital, School of Medicine, Tongji University, Shanghai, China; 2 Department of Orthopedics, Shanghai Baoshan Luodian Hospital, Shanghai, China; 3 Department of Sports Medicine, The Quzhou Affiliated Hospital of Wenzhou Medical University, Quzhou People’s Hospital, Quzhou, Zhejiang, China

**Keywords:** arthroscopic rotator cuff repair, mesenchymal stem cell, regenerative medicine, rotator cuff injury, subacromial bursa, tendon healing, tissue engineering

## Abstract

Rotator cuff injury (RCI) represents a leading cause of shoulder pain and functional impairment, imposing a considerable burden on patients’ quality of life and healthcare systems worldwide. The subacromial bursa (SAB), a critical anatomical structure located between the acromion and rotator cuff tendons, has traditionally been regarded as a primary pathological contributor to pain generation, inflammation, and tendon degeneration. Aberrant inflammatory signaling, fibrotic remodeling, and nociceptive sensitization within the diseased SAB can disrupt the homeostatic tendon microenvironment and impair intrinsic healing capacity, thereby supporting the historical rationale for routine bursectomy during surgical repair. Paradoxically, accumulating evidence challenges this conventional view, suggesting that the SAB also serves as a biologically active, endogenous regenerative reservoir. It contains mesenchymal stem cells (MSCs) with promising proliferative and tenogenic potential, secretes bioactive growth factors, and modulates the local immune microenvironment to promote reparative and anti-inflammatory responses conducive to tendon healing. This dual biological role presents an important clinical dilemma in arthroscopic rotator cuff repair: should surgeons resect pathological bursal tissue to alleviate the inflammatory burden and improve visualization, or selectively preserved to maintain its potential regenerative capacity? In this comprehensive narrative review, we critically synthesize current evidence regarding the pathological and regenerative functions of the SAB in RCI, critically evaluate the ongoing controversy between bursal resection versus preservation, and summarize emerging SAB-targeted therapeutic strategies. Furthermore, we integrate multi-omics insights into bursa biology aimed at moderating the local microenvironment. Finally, we highlight current translational challenges and future directions to inform biologically guided and evidence-based management of the SAB for rotator cuff repair.

## 1 Introduction

Rotator cuff injury (RCI) is a principal cause of shoulder pain and dysfunction, with its incidence rising markedly with age and accounting for approximately 40% of all shoulder disorders (Luime et al., 2004; Yamaguchi et al., 2006; Tashjian, 2012). As a major musculoskeletal disorder, RCI imposes a substantial socioeconomic and healthcare burden, severely diminishing the quality of life and productivity of millions of affected individuals worldwide. In the United States alone, an estimated 17 million individuals are affected, resulting in approximately 500,000 rotator cuff repair procedures annually, and the incidence continues to rise with population ageing (Mather et al., 2013; Marshall et al., 2024). Current clinical management of RCI is broadly divided into conservative and surgical approaches. For mild to moderate injuries, conservative modalities, including physical therapy, oral nonsteroidal anti-inflammatory drugs (NSAIDs), high-energy extracorporeal shockwave therapy (ESWT), and subacromial corticosteroid injections, are widely employed (Walther et al., 2004; Gaujoux-Viala et al., 2009; Huisstede et al., 2011). However, when conservative treatment fails or structural symptoms progress, surgical repair becomes imperative. Despite substantial advances in arthroscopic techniques, postoperative retear rates remain unacceptably high, ranging from 20% to 90%. This high failure rate is largely attributed to poor tendon vascularity, cellular scarcity, and restricted intrinsic regenerative capacity (Galatz et al., 2004; Kim et al., 2013). These persistently high retear rates underscore an urgent need to better understand the local biological microenvironment that constrains tendon healing and functional recovery rather than focusing exclusively on mechanical repair (Wang et al., 2025).

**FIGURE 1:**
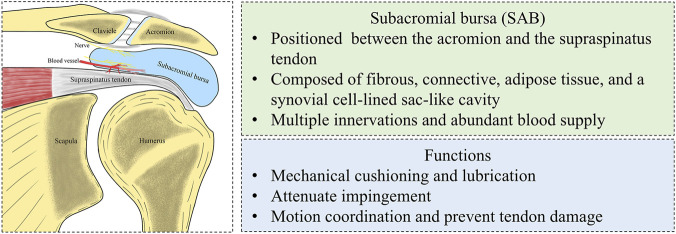
Anatomical and functional landscape of the subacromial bursa (SAB).

The subacromial bursa (SAB) is a specialized synovial, sac-like structure located between the acromion and the rotator cuff tendons (Figure 1), consisting of a synovial cell layer supported by fibrous connective and adipose tissues (Lanham et al., 2021). Physiologically, the SAB serves two principal functions. Mechanically, it cushions and lubricates the subacromial space through the secretion of synovial fluid, thereby reducing friction, attenuating subacromial impingement, and facilitating smooth tendon gliding beneath the coracoacromial arch. Sensorily, the SAB contributes significantly to proprioception, as its richly innervated bursal wall contains mechanoreceptors, including Pacinian and Golgi-Mazzoni corpuscles, which participate in neural feedback mechanisms that regulate shoulder kinematics, enhance motor coordination, and prevent excessive motion-induced injuries (Ide et al., 1996; Tran et al., 2021). The rotator cuff muscles, along with their encircling tendons, collectively facilitate rotational motion and stabilize the glenohumeral joint (Malcarney and Murrell, 2003). Among these structures, the supraspinatus tendon plays a particularly critical role during the initial phase of shoulder abduction and is also the most susceptible to injury (Prescher, 2000). Accordingly, the dynamic buffering interface established by the SAB between the rotator cuff and coracoacromial arch is indispensable for maintaining normal shoulder biomechanics and preserving structural tendon integrity.

Historically, pathological alterations within the SAB are closely associated with the pathogenesis and progression of RCI (Skazalski et al., 2021). The inflamed bursa typically exhibits synovial edema, hyperemia, hyperplasia, subsequent fibrosis, and fatty infiltration (Ko et al., 2019). Traditionally, these pathological changes have implicated the SAB as an important contributor to pain generation and chronic inflammatory progression (Voloshin et al., 2005; Lewis, 2010; Chillemi et al., 2016; Klatte-Schulz et al., 2023). Elevated levels of pro-inflammatory cytokines, including IL-1β, IL-6, and TNF-α, may stimulate local nociceptors and disseminate into adjacent tendon tissues, thereby exacerbating degeneration and impairing tendon healing. Pathological alternations within the bursa may disrupt the local microenvironment and compromise reparative processes because the SAB partially shares vascular supply with the supraspinatus tendon (Voloshin et al., 2005; Wang et al., 2015; Põldoja et al., 2017; HajAssaad et al., 2020). Intraoperatively, hypertrophic, edematous, or fibrotic bursa tissues frequently obscure arthroscopic visualization and restrict maneuverability. Consequently, the widespread use of bursectomy has long been standard clinical practice to improve surgical exposure and eliminate inflammatory burden (Donigan and Wolf, 2011; Lerch et al., 2016). Under this historical framework, the SAB has long been viewed predominantly as a pathological tissue requiring thorough debridement.

Nevertheless, this traditional perspective has been increasingly challenged by emerging biological evidence. Recent studies have successfully isolated functional mesenchymal stem cells (MSCs) from surgically discarded surgical SAB tissue (Muschler and Midura, 2002; Lhee et al., 2013; Levy et al., 2022). These bursal-derived cells exhibit promising proliferative, clonogenic, and multilineage differentiation capacities, including tenogenic potential that, in some experimental research, appears comparable or potentially higher than bone marrow-derived MSCs (Utsunomiya et al., 2013; Dyrna et al., 2018; Kriscenski et al., 2022). In addition, bursal tissue functions as a biologically active reservoir capable of secreting various growth factors involved in angiogenesis, cell proliferation, migration, and extracellular matrix synthesis (Sakai et al., 2001; Yanagisawa et al., 2001; Li et al., 2023). Importantly, accumulating evidence suggests that bursal cells may exert beneficial immunomodulatory effects, notably by facilitating macrophage polarization from the pro-inflammatory M1 to anti-inflammatory M2 phenotype, thereby fostering a more favorable healing microenvironment (Ju et al., 2024). Collectively, these findings strongly indicate that the SAB serves not merely as a passive bystander or pathological contributor, but rather a potentially valuable endogenous resource for tissue regeneration (Uhthoff and Sarkar, 1991). These findings frame the SAB as a tissue with dual and context-dependent biological roles, simultaneously contributing to pathological progression while also retaining regenerative potential. This duality creates a central clinical dilemma in arthroscopic rotator cuff repair: should surgeons routinely resect pathological bursa tissue to improve visualization and alleviate inflammatory burden, or selectively preserved to maintain its potential reparative capacity? Whereas current clinical management for the SAB relies heavily on preclinical findings, there remains limited consensus on which patients would benefit from bursal preservation versus resection. Furthermore, the precision biological transition of the SAB from a predominantly inflammatory tissue to a reparative microenvironment remains incompletely elucidated.

In this comprehensive narrative review, we analyze both degenerative and traumatic rotator cuff injuries, with a particular emphasis on degenerative lesions, given that the current evidence regarding chronic inflammation, fibrotic remodeling, and subacromial bursal pathology is predominantly derived from degenerative conditions. We critically synthesize the dual, context-dependent roles of the SAB as both a pathological contributor and a regenerative resource in RCI. Moreover, we critically evaluate the ongoing controversies regarding bursal resection versus preservation, summarize prevailing translational challenges, highlight emerging therapeutic strategies, and propose future directions for biologically informed and evidence-based rotator cuff repair.

## 2 The pathological contributor

In its pathological state, the SAB has traditionally been characterized as an important contributor to rotator cuff injury progression, operating as a biologically active induction of inflammation, pain sensitization, and tissue degeneration (Budoff et al., 2005). Substantial evidence indicates significantly elevated levels of multiple pro-inflammatory cytokines and enzymatic mediators within the SAB of patients with RCI, most notably interleukin-1β (IL-1β), interleukin-6 (IL-6), tumor necrosis factor-α (TNF-α), and cyclooxygenase-2 (COX-2) (Blaine et al., 2011; Lho et al., 2013; Minkwitz et al., 2021). The upregulation of these mediators correlates tightly with clinical symptom severity, including persistent pain, joint stiffness, and restricted shoulder motion. Critically, inflammatory mediators synthesized within the bursa can diffuse into adjacent tendons via paracrine pathways, where they induce tenocyte apoptosis (Schulze-Tanzil et al., 2011; Osti et al., 2017). Moreover, this paracrine signaling activates proteolytic enzymes, thereby accelerating extracellular matrix (ECM) catabolism and inhibiting collagen synthesis (Del Buono et al., 2012; Uehara et al., 2023). Collectively, these processes may compromise tendon structural integrity and impair its intrinsic healing capacity. Additionally, under pathological conditions, bursa-derived bone morphogenetic proteins have been implicated in driving aberrant cartilage formation and ectopic ossification (Neuwirth et al., 2006).

Importantly, the biological significance of inflammation within the SAB is not uniformly detrimental but is highly stage-dependent and context-specific. During the acute phase of RCI, transient inflammatory signaling may facilitate debris clearance, local angiogenesis, immune-cell recruitment, and initial ECM remodeling, thereby supporting early tissue repair. However, when these inflammatory signals become persistent or dysregulated, prolonged macrophage activation, sustained cytokine secretion, and oxidative stress may progressively shift the local microenvironment toward chronic pathological remodeling rather than tissue regeneration, including excessive ECM deposition, nociceptive sensitization, and tissue degeneration (Hyvönen et al., 2003). Furthermore, pathological changes such as fatty infiltration, fibrotic destruction, and abnormal angiogenesis are common in the diseased bursa (Kronberg and Saric, 1997; Lewis, 2009; Baldino et al., 2020). These structural alterations compromise the physiological utility of the SAB. A fibrotic, non-compliant bursa fails to provide adequate lubrication and cushioning, thereby increasing mechanical friction during humeral elevation and exacerbating subacromial impingement. Simultaneously, the structural disruption of mechanoreceptor signaling within the diseased bursa wall impairs joint proprioception and compromises neuromuscular coordination (Kennedy et al., 2022). Moreover, pathological bursal remodeling may disrupt the shared vascular microenvironment network supplying the supraspinatus tendon, potentially exacerbating ischemic degeneration and further diminishing the tendon’s intrinsic regenerative potential (Gumina et al., 2006; Põldoja et al., 2017).

**FIGURE 2:**
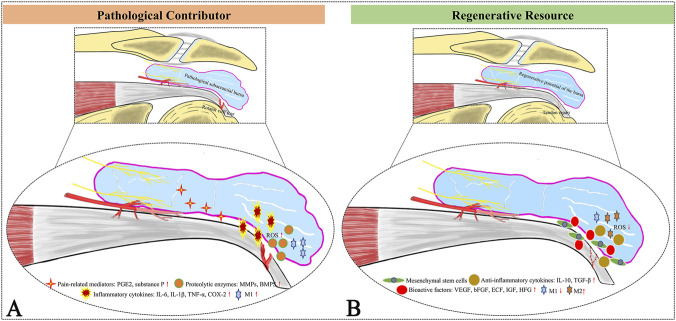
The biological duality of the SAB in rotator cuff pathology and tissue healing. **(A)** Pathological driver. **(B)** Regenerative engine.

From an anatomical perspective, the SAB is densely innervated by branches of the suprascapular, lateral pectoral, and axillary nerves, which sharply contrasts with the relatively sparsely innervated coracoacromial ligament (Nasu et al., 2015; Laumonerie et al., 2019). The bursal wall contains abundant nociceptive free nerve endings expressing pain-related receptors, establishing its role as a primary source of pain in RCI (Park et al., 2020). During shoulder movement, the thickened and fibrotic bursal tissue may subject these nerve endings to abnormal compression and traction. Concurrently, local inflammatory mediators, alongside biochemical pain-related mediators including prostaglandin PGE2 and substance P, may sensitize sensory endings and trigger persistent activity-related pain (Gotoh et al., 1998; Yoon et al., 2025). Over time, a self-perpetuating pathological cycle may develop, in which rotator cuff tears and subacromial impingement exacerbate bursal inflammation, while this persistent bursal pathology further amplifies clinical pain and functional limitation (Lewis, 2009). These pathological mechanisms provide an important historical rationale for bursectomy during rotator cuff repair (Freislederer et al., 2019). However, such a conventional approach focuses exclusively on these detrimental facets, potentially neglecting the valuable, reparative functions intrinsic to this tissue (Figure 2).

## 3 The regenerative resource

In contrast to the traditional paradigm viewing the SAB as a predominantly pathological tissue, emerging evidence underscores its capacity to act as a critical endogenous resource for tendon regeneration during rotator cuff healing (Gregory et al., 2023). Rather than being a passive bystander, the SAB possesses a dynamic reparative potential that contributes to tissue regeneration through multiple complementary mechanisms, including the provision of endogenous stem cells, the secretion of bioactive factors, and the modulation of the local immune microenvironment.

One of the most extensively investigated regenerative features of the SAB is its resident reservoir of MSCs. Multiple studies have successfully isolated MSCs from SAB tissue and demonstrated their multilineage differentiation potential (Morikawa et al., 2021b; Marshall et al., 2023). When compared to BMSCs, SAB-derived MSCs (SAB-MSCs) display promising proliferative and tenogenic differentiation capacities (Tamburini et al., 2021). Moreover, SAB-MSCs exhibit several distinct biological and clinical advantages to support their regenerative relevance. First, these cells express tendon-related genes, including Scleraxis (Scx) and Tenomodulin (Tnmd), which may facilitate tendon-specific differentiation (Wang et al., 2023; Marshall et al., 2024). Second, they actively drive extracellular matrix synthesis through the increased production of Type I/III collagen alongside tendon-specific extracellular matrix components (Yoshida et al., 2016; Morikawa et al., 2019). Third, unlike BMSCs that require invasive bone marrow aspiration, SAB-MSCs can be seamlessly harvested intraoperatively during arthroscopic procedures without additional invasive trauma and donor-site morbidity, providing an important practical advantage for clinical translation (Morikawa et al., 2020; Morikawa et al., 2021a). Owing to inherent accessibility and robust biological potency, SAB-MSCs stand out as promising candidates for targeted rotator cuff regenerative therapies. To further elaborate on its superiorities over other common seed cell sources, the core biological features of SAB-MSCs, BMSCs, and tendon-derived stem cells (TDSCs) are contrasted in Table 1.

**TABLE 1:** Comparative biological characteristics of subacromial bursa-derived MSCs (SAB-MSCs), bone marrow-derived stem cells (BMSCs), and tendon-derived stem cells (TDSCs).

Characteristics	SAB-MSCs	BMSCs	TDSCs
Tissue source	Subacromial bursa	Iliac crest/bone marrow aspirate	Native rotator cuff tendon
Harvesting invasiveness/Clinical accessibility	Low/Easily (harvested during operation)	High/limited (additional procedure required)	High/limited (tendon tissue-consuming)
Cellularity	Moderate to high	High	Moderate
Proliferation potential	High to moderate	High	Moderate
Tenogenic differentiation	High	Moderate to high	High
Chondrogenic differentiation	Moderate to high	High	Moderate
Osteogenic differentiation	Moderate	High	Moderate
Immunomodulatory properties	Strong	Strong	Moderate
Adaptation to the tendon microenvironment	Potentially favorable (native shoulder environment)	Moderate to high	High (derived from native tendon)

Nonetheless, the ultimate regenerative competence of SAB-MSCs likely depends on the biological state of the surrounding microenvironment. Chronic fibrotic remodeling, persistent inflammatory infiltration, oxidative, and mechanical bursa stiffening can severely impair stem cell viability, migratory behavior, and differentiation potential. Beyond serving as a cellular reservoir, the SAB actively influences the reparative microenvironment through the secretion of diverse bioactive molecules involved in tissue remodeling and healing. Several growth factors implicated in tendon healing have been identified within bursal tissue. For instance, vascular endothelial growth factor (VEGF) facilitates early-stage repair by stimulating angiogenesis, tissue perfusion, and metabolic clearance (Yanagisawa et al., 2001); Basic fibroblast growth factor (bFGF) has been implicated in fibroblast proliferation, migration, and collagen synthesis (Sakai et al., 2001); whereas transforming growth factor-β1 (TGF-β1) contributes to ECM remodeling and structural integrity of healing tendon (Li et al., 2023). Furthermore, epidermal growth factor (EGF), insulin-like growth factor (IGF), hepatocyte growth factor (HGF), and anti-inflammatory mediators secreted by the SAB further regulate cellular metabolism, matrix turnover, and reparative signaling (Muench et al., 2021a). Importantly, these signaling pathways function cooperatively within a multifactorial biochemical network that modulates inflammatory responses and shapes the healing response.

A pivotal yet often overlooked function of SAB biology is its capacity to regulate local immune homeostasis. Accumulating literature indicates that SAB-derived signaling molecules can facilitate macrophage polarization from a pro-inflammatory M1 phenotype to a pro-repair M2 phenotype (Chillemi et al., 2011; Wang et al., 2023). While macrophage polarization with M1-dominant responses favors chronic inflammation, M2-associated signaling may promote tissue remodeling and regenerative repair. M2 polarization can enhance the secretion of anti-inflammatory mediators such as IL-10 and TGF-β, accelerate the clearance of apoptotic cell debris, and ameliorate local oxidative stress (Sakai et al., 2001). Simultaneously, extracellular matrix secreted by activated fibroblasts, including collagen and fibronectin, may provide a provisional mechanical scaffold that influences cell migration and phenotypic regulation through integrin-associated signaling pathways (Tamburini et al., 2021). These immune and stromal components establish a microenvironment more conducive to functional tissue regeneration. Accordingly, the SAB is increasingly recognized as a dynamically dual-functional entity during RCI progression, actively contributing to inflammation while preserving intrinsic regenerative potential. This functional duality underlines the long-standing clinical controversy over optimal bursal management: the trade-off between bursal bursectomy to alleviate acute symptoms and selective bursal preservation to safeguard an invaluable endogenous regenerative engine.

## 4 The clinical dilemma: to resect or preserve the SAB?

**FIGURE 3:**
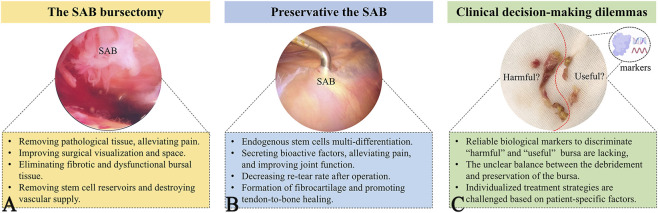
Clinical considerations for SAB management in arthroscopic rotator cuff repair. **(A)** Rationale and potential consequences of bursectomy. **(B)** Potential advantages of bursal preservation. **(C)** Current evidence gap.

The optimal management of the SAB during rotator cuff repair remains controversial, largely due to its dual, context-dependent biological role. Historically, total bursectomy has been routinely performed to optimize surgical visualization, eliminate the local inflammatory tissue burden (IL-1β, IL-6, and TNF-α) driving tendon destruction, and alleviate pain associated with bursal inflammation and dense nociceptive innervation (Voloshin et al., 2005; Lho et al., 2013). However, researchers have discovered that as healing progresses, the SAB may partially transition toward a reparative mode through the activation of resident endogenous MSCs, the secretion of pro-repair factors, and the induction of M2 macrophage polarization (Lhee et al., 2013; Song et al., 2014). Conversely, chronic inflammation and excessive fibrosis can disrupt this homeostatic balance, ultimately impairing MSC function and overall healing capacity (Klatte-Schulz et al., 2022). Pathological SAB tissue presents significant technical challenges during arthroscopic rotator cuff repair (ARCR) over biological considerations. Hypertrophic bursa tissue frequently obscures visualization and may impair accurate assessment of tendon morphology (Gotoh et al., 1998; Ko et al., 2019). Furthermore, extensive bursal proliferation may complicate instrument maneuverability and interfere with bone-bed preparation and anchor placement with shavers and radiofrequency devices. These mechanical factors potentially affect procedural efficiency, compromise repair quality, and prolong operative time. Consequently, routine bursectomy has long been favored to improve visualization, relieve postoperative pain, and facilitate surgical workflow (Klatte-Schulz et al., 2022). Nevertheless, the indiscriminate removal of bursal tissue inherently sacrifices several major biological advantages, including eliminating a potentially valuable reservoir of stem cells, disrupting the secretion of growth factors, and altering the vascular and mechanical microenvironment shared with the supraspinatus tendon (Figure 3). Therefore, the traditional bursectomy paradigm may warrant reconsideration in light of emerging regenerative evidence.

Experimental strategies investigating bursal preservation remain promising yet preliminary (Muench et al., 2020). Emerging preclinical animal studies suggest that the preservation or autologous reimplantation of SAB tissue can significantly enhance tendon-to-bone healing improve collagen organization, and increase biomechanical strength (Lebaschi et al., 2022). Similarly, several clinical studies have reported that retaining or even implanting SAB tissue during arthroscopic repair correlates with lower retear rate, reduced pain, and improved functional outcomes (Muench et al., 2021b; Gregory et al., 2023). However, these findings must be interpreted cautiously, as much of the available evidence is restricted to observational, retrospective, and preclinical experiments, lacking high-quality prospective studies and randomized clinical trials for validation. Despite growing interest in biologically informed preservation strategies, several important translational barriers remain unresolved. First, intraoperative evaluation of bursal quality remains largely subjective and currently relies heavily on gross appearance, including tissue color, texture, and degree of fibrosis. Reliable biological markers to differentiate “harmful” from “useful” SAB tissue are still lacking (Baldino et al., 2020). Future advances may require an objective assessment system incorporating imaging biomarkers, molecular staining, or real-time cytokine profiling. Second, the precise balance between selective debridement and tissue preservation remains undefined. Novel approaches, including selective partial bursectomy or vascularized bursal flap techniques, may offer strategies to retain biological benefits while minimizing pathological burden (Bhatia, 2021). Third, patient-specific stratification remains poorly defined. Factors including injury chronicity, tear size, age, metabolic status, and degree of fibrosis may influence which patients are more likely to benefit from preservation versus resection, although definitive evidence is currently lacking. For instance, elderly patients with retracted, chronic tears and severely fibrotic bursae may benefit from thorough resection. Conversely, younger, active individuals with acute traumatic tears and a robust, viable bursal tissue may benefit from bursal preservation.

**FIGURE 4:**
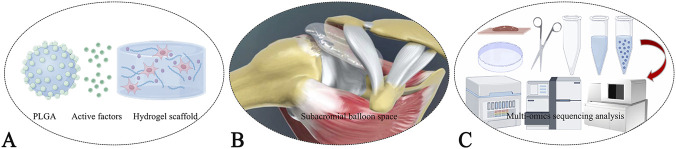
Emerging and precision-based strategies for targeted SAB management. **(A)** Localized therapeutic delivery: Schematic representation of advanced drug delivery system, such as biodegradable PLGA microspheres or hydrogel scaffolds, engineered for the sustained release of bioactive agents within the bursal region (created with Figdraw). **(B)** Biomechanical augmentation via subacromial spacer: *In situ* illustration of a balloon-shaped implant (e.g., InSpace device) deployed in the subacromial space to reduce friction and pain (figure was adapted from Stryker’s official website). **(C)** Precision medicine pathway: A proposed framework integrating multi-omics profiling of bursal tissue to guide individualized surgical and pharmacological interventions (created with Figdraw).

Contemporary evidence increasingly challenges the traditional view of the SAB as merely pathological tissue while simultaneously cautioning against overestimating its regenerative benefits. Rather than routine excision or indiscriminate preservation, future management strategies may shift toward biologically informed and patient-specific modulation of the SAB. Such an approach may enable suppression of maladaptive inflammatory responses while preserving intrinsic regenerative capacity, ultimately spawning a series of more precise, innovative regenerative interventions for the SAB (Figure 4).

## 5 Innovative therapeutic strategies for modulating the SAB

Given the dual pathological and regenerative nature of the SAB, therapeutic strategies are increasingly emphasizing selective biological modulation rather than indiscriminate tissue removal. Instead of simply excising pathological tissue, recent approaches aim to suppress maladaptive inflammation while preserving or augmenting the SAB’s intrinsic reparative capacity. Conceptually, these emerging approaches can be broadly categorized into the following domains: anti-inflammatory modulation, SAB-MSCs augmentation and bioactive molecules, biomaterials and scaffold-based approaches, and exosome-mediated interventions.

### 5.1 Anti-inflammatory modulation

Because persistent inflammatory activation contributes to chronic bursal remodeling and subsequent tendon degeneration, modulation of the local inflammatory microenvironment represents a rational therapeutic target. Unlike systemic anti-inflammatory treatment, localized intervention within the subacromial space may offer greater biological precision while minimizing adverse effects. The unique anatomical architecture and adipose-rich properties of the SAB make it an ideal natural depot for localized and sustained drug delivery (Huebner et al., 2014). Biodegradable carriers, including PLGA microspheres and nanoparticles, have been explored for encapsulation of anti-inflammatory agents (e.g., dexamethasone). This localized delivery architecture optimizes pharmacokinetics, effectively inhibiting pro-inflammatory cytokines and nitric oxide (NO) secretion while minimizing systemic adverse effects associated with repeated corticosteroid administration (Marshall et al., 2024). In parallel, increasing attention has focused on targeted molecular interventions on inflammatory signaling pathways. For example, transforming growth factor-activated kinase 1 (TAK1) inhibitors have been proposed as a strategy to attenuate IL-1β-mediated inflammation via the p38 MAPK pathway, thereby downregulating pain-associated mediators such as nerve growth factor (NGF) and cyclooxygenase-2 (COX-2) (Nagura et al., 2019; Tazawa et al., 2021). Additionally, immune-directed approaches designed to drive macrophage polarization toward a reparative phenotype through IL-4/JAK/STAT signaling axis have demonstrated encouraging outcomes in accelerating fibrocartilaginous tissue remodeling and tendon-to-bone healing (Liu et al., 2022).

### 5.2 SAB-MSCs augmentation and bioactive molecules

In addition to controlling inflammation, regenerative strategies increasingly seek to augment the endogenous reparative potential of the SAB. Owing to their immediate intraoperative accessibility during arthroscopic surgery and their promising proliferative and tenogenic differentiation capacities, SAB-MSCs represent a promising regenerative candidate for biologically augmented rotator cuff repair (Morikawa et al., 2019; Morikawa et al., 2020). These cells can be expanded *ex vivo* and subsequently delivered to the tendon-bone interface using biocompatible scaffolds, injectable hydrogels, or cell-loaded biomaterials to promote tissue regeneration (Zhang et al., 2024). In parallel, less invasive alternative strategies focus on the *in situ* pharmacological activation of resident SAB-MSCs. Delivering targeted bioactive molecules, including small-molecule agonists or bone morphogenetic protein-7 (BMP-7) through controlled-release gelatin hydrogel sheet, has shown immense potential in stimulating endogenous stem cell migration and tissue repair (Kabuto et al., 2015). Nevertheless, clarifying the long-term biological stability, establishing optimal delivery vectors, and ensuring the reproducibility of SAB-MSCs therapies remain incompletely understood and require further investigation.

### 5.3 Biomaterials and scaffold-based approaches

Biomaterial-assisted strategies represent a powerful avenue for restoring the biomechanical and biological functions of diseased SAB, particularly in patients with irreparable or massive rotator cuff tears. Advanced synthetic biomaterials partially recapitulate the mechanical cushioning and physiological functions of the native SAB (Savarese and Romeo, 2012; Moon et al., 2019; Oh et al., 2019). Among currently available approaches, biodegradable subacromial balloon spacers have attracted considerable clinical interest. Typically composed of a biodegradable poly-L-lactide-eco-ε-caprolactone copolymer, these devices are designed to mimic the bursa’s role in separation and lubrication, reducing acromiohumoral friction, and improving biomechanics during movement. Following implantation, the spacers are gradually resorbed over approximately 12 months and replaced by functional fibrous tissue (Srikumaran et al., 2023). The future of biomaterial development may move beyond purely mechanical support toward biologically active scaffolds. For example, next-generation “bioactive spacer” capable of releasing anti-inflammatory compounds, antioxidant scavengers, or pro-regenerative molecules can simultaneously provide immediate biomechanical relief while fostering an inductive microenvironment for long-term tissue regeneration. However, optimizing scaffold degradation, ensuring biomechanical compatibility, and verifying long-term biological safety remain significant engineering challenges.

### 5.4 Exosome-mediated interventions

Compared with cell transplantation, exosome-based therapies provide a compelling cell-free regenerative alternative with low immunogenicity and fewer safety concerns. A key mediator of intercellular communication, exosomes derived from SAB-MSCs have demonstrated a powerful ability to regulate the tendon-to-bone healing microenvironment by reducing inflammatory cell infiltration and orchestrating macrophage polarization toward reparative M2 phenotypes (Zou et al., 2023). In addition, intelligent nanocarriers (e.g., liposomes or polymeric nanoparticles) can be engineered to encapsulate and deliver bioactive factors, including Platelet-Rich Plasma (PRP), VEGF, bFGF, and TGF-β, thereby remodeling the local immune and regenerative niche of the SAB (Muench et al., 2023; Marshall et al., 2024). Although these exosome-mediated therapies show considerable promise, challenges, including manufacturing standardization, dosing optimization, storage stability, and regulatory approval, remain significant barriers to widespread clinical application (Morikawa et al., 2019; Morikawa et al., 2020).

These emerging therapeutic strategies reflect a fundamental conceptual shift in the understanding of the SAB in RCI, moving beyond conventional bursa bursectomy or preservation toward modulation of the SAB microenvironment. Nevertheless, no single intervention can fully resolve such intricate biological complexity. Further advances will rely on combinatorial strategies that integrate immune regulation, biomaterial engineering, and regenerative medicine, while accounting for the significant dynamic fluctuations in the molecular signatures, metabolism profiles, and protein dynamics within SAB throughout RCI progression.

## 6 Multi-omics insights: toward precision modulation of the SAB microenvironment

Recent breakthroughs in transcriptomics, proteomics, metabolomics, and single-cell sequencing have profoundly expanded our comprehension of the biological complexity and cellular heterogeneity inherent to the SAB microenvironment. Rather than functioning solely as an inflammatory tissue, the SAB exhibits highly dynamic molecular signatures that vary according to inflammatory status, injury chronicity, fibrosis severity, and patient-specific characteristics. These emerging multi-omics approaches provide new opportunities to reshape the understanding of SAB pathology, shifting from a histologically defined tissue abnormality toward a molecularly stratified, clinically targetable biological therapeutic modality.

Transcriptomic investigations have successfully identified differential molecular signatures associated with inflammation, fibrosis, extracellular matrix (ECM) remodeling, and tissue regeneration within the bursa. In patients with pathological bursa, dysregulated inflammatory pathways, including IL-7 signaling, immune-cell activation, and altered ECM receptor interactions, appear closely associated with chronic bursitis and adhesive shoulder pathology (Yuan et al., 2021). Simultaneously, multiple hub genes central to matrix remodeling, such as MMP2/9, IL-6, and COL1A1, are frequently upregulated, suggesting active tissue remodeling and chronic inflammatory activation within the diseased SAB (Wang et al., 2024). Interestingly, the downregulation of MMP1/3 observed in some pathological settings further highlights the complexity of matrix remodeling dynamics rather than a uniformly destructive process.

Differential transcriptomic signatures revealed that the molecular landscape of the SAB is highly sensitive to patient sex and injury etiology (Rai et al., 2022). In female patients, traumatic tears are characterized by the activation of muscle cell differentiation, development, and contraction pathways, whereas degenerative tears are dominated by immune cell responses. Conversely, male patients exhibit an enrichment in energy metabolism pathways during acute traumatic tears, while cell-cycle transitions prevail in chronic degenerative cases. These findings underscore the possibility that SAB biology differs substantially among clinical patient subgroups, highlighting the absolute necessity for biologically informed and individualized therapeutic approaches.

Beyond transcriptomics, proteomic analyses further reinforce the concept that the SAB exhibit both pathological and protective biological functions. For instance, while immune-derived chemokines and inflammatory mediators released by activated macrophages and dendritic cells can exacerbate chronic inflammation (Ju et al., 2024), they can also impede recovery by dysregulating the proliferation and differentiation of MSCs, potentially hindering the repair and regeneration of injured tissue (Handa et al., 2003). Notably, superoxide dismutase 3 (SOD3) has been implicated in oxidative stress defense and DNA damage repair, potentially protecting adjacent structures such as the infraspinatus tendon and humeral head from oxidative injury (Marshall et al., 2024).

The computational integration of transcriptomic and proteomic signatures holds immense promise for predicting healing potential, identifying patients at higher risk of fibrosis or retear, and supporting the development of more personalized regenerative interventions. However, the current clinical feasibility of these precision-based approaches remains limited by high cost, technical complexity, a lack of standardized molecular platforms, and insufficient prospective validation. Future large-scale clinical trials correlating longitudinal multi-omics datasets with clinical outcomes are imperative. Such endeavors will facilitate molecular subtyping, optimize prognostic assessments, and ultimately empower clinicians to determine whether selective preservation, modulation, or resection is the most biologically appropriate course of action for individual patients.

## 7 Translational challenges and future perspectives

Although increasing evidence highlights the biological relevance of the SAB in rotator cuff healing, successful clinical translation into routine clinical practice remains in its infancy. A major challenge lies in safely reconciling the SAB’s dual pathological contributor and regenerative resource. Local inflammatory burden, fibrosis severity, vascular integrity, and regenerative potential may vary substantially according to tear chronicity, injury etiology, and patient-specific factors. Consequently, identifying which patients will derive the greatest benefit from bursal preservation, selective modulation, or resection remains poorly defined.

Another important limitation is the quality and consistency of the current evidence base. Encouraging findings are predominantly composed of *in vitro* experiments, animal models, and retrospective clinical analyses with small sample sizes. While these studies provide valuable mechanistic insights, they incompletely recapitulate the anatomical complexity and biomechanical heterogeneity encountered in human rotator cuff repair. High-quality, multi-center prospective cohorts and randomized clinical trials are therefore required to determine the safety, efficacy, and standardize SAB-targeted interventions.

Future breakthroughs will inevitably depend on the integration of biologically informed surgical strategies with advances in regenerative medicine and precision molecular profiling. Incorporating high-throughput multi-omics approaches will help to identify SAB’s molecular, inflammation, fibrotic, and regenerative phenotypes, thereby supporting more individualized intraoperative decision-making. Simultaneously, development of biologically responsive biomaterials and targeted immunomodulatory interventions will enable more precise regulation of the bursal microenvironment while preserving endogenous reparative potential.

## 8 Conclusion

The subacromial bursa (SAB) occupies a paradoxical yet pivotally important position in rotator cuff pathology and repair. Though historically deemed a primary culprit of local inflammation, pain generation, and surgical obstruction, the SAB has been redefined as a functionally dynamic tissue with pathological and regenerative properties. Its functional behavior appears highly context-dependent and influenced by inflammatory status, fibrosis severity, cellular composition, injury chronicity, and local microenvironmental conditions.

This intrinsic biological duality drives a central clinical dilemma in rotator cuff repair. While excessive inflammation, fibrosis, and nociceptive signaling warrant mechanical debridement, indiscriminate bursectomy risks inadvertently discarding an invaluable source of MSCs, reparative signaling molecules, and immunomodulatory activity. Consequently, the therapeutic paradigm is decisively swinging away from routine tissue excision toward biologically informed modulation of the SAB microenvironment. Approaches including selective preservation, cell-based augmentation, biomaterial-assisted regeneration, exosome therapies, and multi-omics guided precision strategies may collectively expand future treatment possibilities. Future research should prioritize deeper characterization of SAB microenvironmental dynamics, development of biologically responsive therapeutic platforms, and establishment of reliable intraoperative assessment systems capable of distinguishing pathological from reparative bursal states. Ultimately, a deeper understanding of SAB biology may facilitate more precise and individualized therapeutic strategies, transforming the SAB from a historically neglected tissue into a clinically actionable target for biologically optimized tendon healing.
